# Root Exudates from Grafted-Root Watermelon Showed a Certain Contribution in Inhibiting *Fusarium oxysporum* f. sp. *niveum*

**DOI:** 10.1371/journal.pone.0063383

**Published:** 2013-05-20

**Authors:** Ning Ling, Wenwen Zhang, Dongsheng Wang, Jiugeng Mao, Qiwei Huang, Shiwei Guo, Qirong Shen

**Affiliations:** 1 Agricultural Ministry Key Lab of Plant Nutrition and Fertilization in Low-Middle Reaches of the Yangtze River, Jiangsu Key Lab and Engineering Center for Solid Organic Waste Utilization, Nanjing Agricultural University, Nanjing, Jiangsu, China; 2 Nanjing Institute of Vegetable Science, Nanjing, Jiangsu, China; Centro de Investigación y de Estudios Avanzados, Mexico

## Abstract

Grafting watermelon onto bottle gourd rootstock is commonly used method to generate resistance to *Fusarium oxysporum* f. sp. *niveum* (FON), but knowledge of the effect of the root exudates of grafted watermelon on this soil-borne pathogen in rhizosphere remains limited. To investigate the root exudate profiles of the own-root bottle gourd, grafted-root watermelon and own-root watermelon, recirculating hydroponic culture system was developed to continuously trap these root exudates. Both conidial germination and growth of FON were significantly decreased in the presence of root exudates from the grafted-root watermelon compared with the own-root watermelon. HPLC analysis revealed that the composition of the root exudates released by the grafted-root watermelon differed not only from the own-root watermelon but also from the bottle gourd rootstock plants. We identified salicylic acid in all 3 root exudates, chlorogenic acid and caffeic acid in root exudates from own-root bottle gourd and grafted-root watermelon but not own-root watermelon, and abundant cinnamic acid only in own-root watermelon root exudates. The chlorogenic and caffeic acid were candidates for potentiating the enhanced resistance of the grafted watermelon to FON, therefore we tested the effects of the two compounds on the conidial germination and growth of FON. Both phenolic acids inhibited FON conidial germination and growth in a dose-dependent manner, and FON was much more susceptible to chlorogenic acid than to caffeic acid. In conclusion, the key factor in attaining the resistance to *Fusarium* wilt is grafting on the non-host root stock, however, the root exudates profile also showed some contribution in inhibiting FON. These results will help to better clarify the disease resistance mechanisms of grafted-root watermelon based on plant-microbe communication and will guide the improvement of strategies against *Fusarium*-mediated wilt of watermelon plants.

## Introduction

Watermelon (*Citrullus lanatus* (Thunb.) Matsum and Nakai) is a widely cultivated fruit that is consumed globally. However, the growth of this plant is often threatened by *Fusarium* in the soil, which causes wilting. *Fusarium*-mediated wilting of watermelon is caused by the pathogen *Fusarium oxysporum* f. sp. *niveum* (FON), which is very prevalent in countries in which farm land has been limited and farmers have been forced to grow the same crop over successive years, such as China. FON is considered the most important soil-borne facultative parasite, leads to economically important losses of watermelon, and limits production in many areas of the world. Once a buildup of FON occurs in the soil, it is difficult to eliminate [1], [2].

Many methods have been employed to overcome FON, such as crop rotation, solarization, chemical fungicides, resistant cultivars, biological control and grafting [3], [4], [5], [6]. Among the methods used in controlling the soil-borne disease, grafting onto resistant rootstocks has been highly effective and is a routine technique in continuous cropping systems in many countries [7]. To date, grafts have been used to induce resistance to both low and high temperatures, enhance nutrient uptake, increase synthesis of endogenous hormones, improve water use efficiency, reduce uptake of persistent organic pollutants from agricultural soils, raise salt and flooding tolerance, and limit the negative effects of boron, copper and cadmium toxicity [8]. In China, approximately 20% of the watermelon crop has been grafted for avoidance of soil pathogens [7]. Previous studies on grafting watermelon onto rootstock have been mostly concerned with the survival rate, the product quality and the effect of disease resistance [9], [10], [11], [12]. Nevertheless, no comprehensive published data are available concerning the effects of grafted watermelon on resistance to the soil-borne pathogen FON.

Root exudation has been suggested to play a central role in influencing interactions with neighboring plants and microbes [12], [13]. The quantity and quality of root exudates depends on plant species, cultivar, developmental stage and environmental factors. A small change in root exudates may lead to large alterations in the population of microorganisms in the rhizosphere [14], [15]. Many plant species are found to resist potential soil-borne pathogens by releasing allelochemicals to the rhizosphere in root exudates [2]. There are some reports on allelopathic suppression exhibited by the root exudates from grafted eggplants against *Verticillium dahliae* [6], [16]. However, little is known about the effect of the allelochemicals in root exudates of grafted watermelon on FON in the rhizosphere. We hypothesized that root exudates of watermelon after grafting, especially the compositions and contents of the allelochemicals, may shift and therefore lead to some contribution in suppressing FON.

To test this hypothesis, a recirculating hydroponic culture system was employed to collect the root exudates. As the bottle gourd (*Lagenaria siceraria* Standl.) has been commonly used as a source of rootstock for watermelon in China [17], the overall objective of this research was to determine whether the root exudates of watermelon are changed by bottle gourd rootstock and increase resistance to *Fusarium*. Because phenolic acids are considered to be signal molecules or allelochemical attractants in root exudates [1], [18], [19], we also investigated whether the changes depended on phenolic compounds and identified some potential phenolic compounds that may enhance resistance. These results will enrich our understanding of disease resistance mechanisms from the perspective of plant-microbe interactions and extend the application of grafting.

## Materials and Methods

### Plant Materials

Watermelon [*Citrullus lanatus* (Thunb.) Matsum. and Nakai var. *Zaojia 8424*] and bottle gourd [*Lagenaria siceraria* (Molina) Standl. var. *dayehuzi*] seeds, obtained from the Nanjing Institute of Vegetable Science, Nanjing, China, were sown in a mixture of Turface and soil in the greenhouse at the Provincial Key Lab of Organic Solid Waste Utilization, Nanjing Agricultural University, Jiangsu, with a 14 h photoperiod at temperatures ranging from approximately 22°C to 30°C and ambient relative humidity. A half-strength Hoagland’s nutrient solution was used daily to irrigate plants after germination.

Grafted-root watermelon seedlings were obtained by grafting watermelon onto bottle gourd rootstocks. Watermelon seedlings with at least one true leaf were grafted using one cotyledon or the slant graft method [7]. To facilitate the union of rootstock and scion, seedlings were placed in a shaded plastic tunnel with a humidifier to maintain 100% humidity and temperatures approximately 28°C for a period of 7–10 d, followed by acclimation to the natural conditions of the greenhouse for 7 d.

### Fungal Isolation and Conidia Preparation

FON (coded NJAUS-1) was isolated from infected watermelon in a greenhouse plot and provided by the Laboratory of plant–microbe interactions, Nanjing Agricultural University, China [20].

FON conidia were prepared by growing plate cultures on potato dextrose agar medium (PDA) at 28°C for 10 days in the dark to induce sporulation as previously described [21]. Plates were drenched with sterile distilled water, and spores were carefully freed from the culture surface with a fine artist’s brush. Afterward, the suspension was filtered through three layers of sterile cheesecloth to eliminate mycelial fragments. The conidia concentration was determined using a hemacytometer.

### Root Exudates Collection

**Figure 1:**
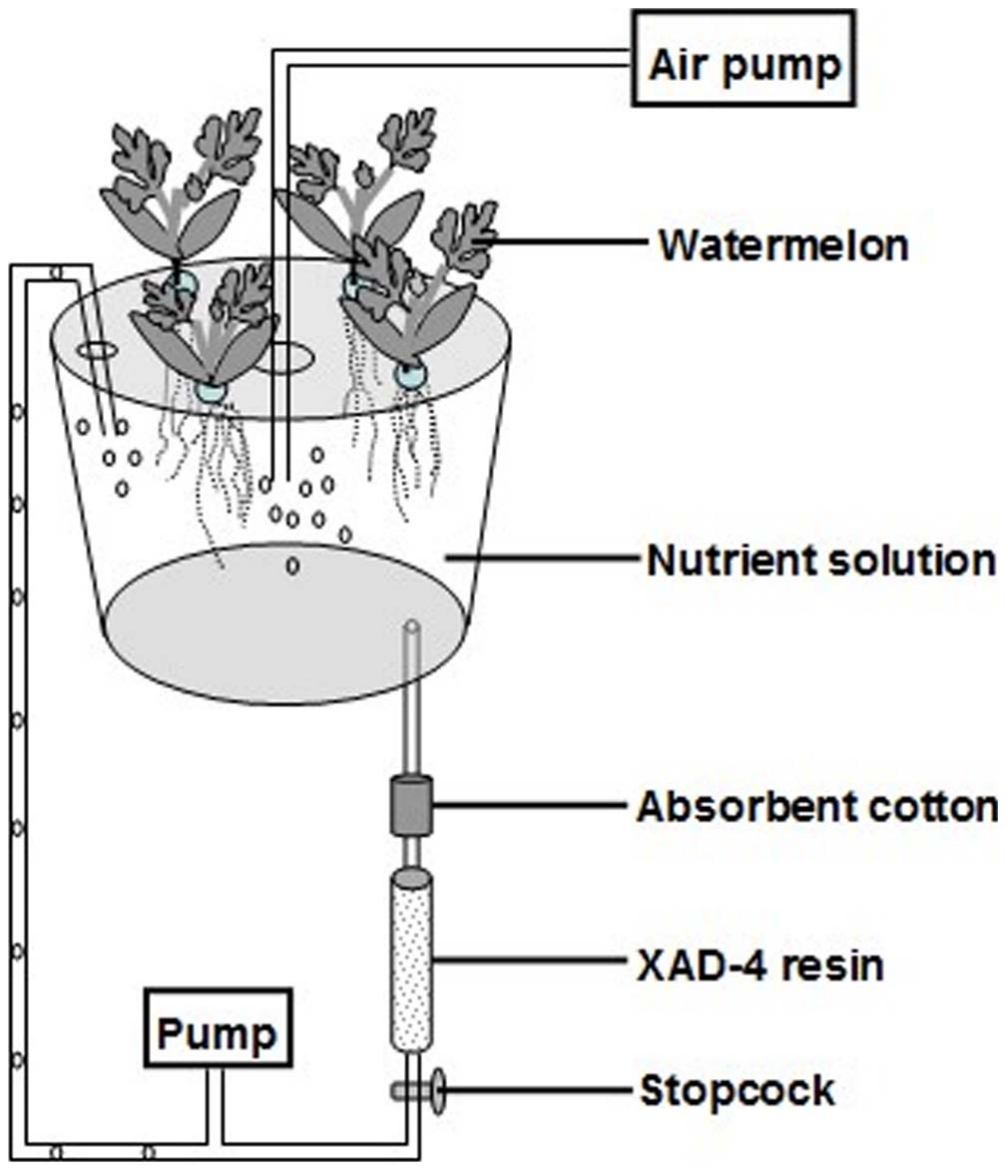
Illustration of the re-circulating hydroponic culture system to continuously trap root exudates. The system was modified according to Hao et al [22].

The three types of seedlings (own-root watermelon, grafted-root watermelon and own-root bottle gourd) at the 5–6 leaf stage were cleaned with deionized water and transplanted into a continuous root exudate trapping system (CRETS) (Fig. 1). The system was designed based on the report by Hao et al. [22] with modifications. The CRETS contained a trapping column that was filled with 10 ml XAD-4 resin (Sigma, USA). The organic compounds released from root exudates into the nutrient solution could be trapped in the column. Above the trapping column, we set a filter column containing cotton wool to remove the root particles in the nutrient solution. The trapping columns were replaced every 2 days. Four seedlings were placed in a 5-L plastic vessel containing hydroponic culture solution, and every type of seedling was placed in three plastic vessels, which served as three replications. The nutrient solution formula described in our previous report [23] was prepared using deionized water and changed every 7 days. The pH of the nutrient solution was measured daily and maintained at 6.0 to ensure that no substantial changes of the nutrient solution occurred throughout the experiment. Plants were grown in the plastic vessels for 30 days in the greenhouse. After 30 days, the resin were eluted with 200 ml of spectral-grade methanol (Sigma, American) and evaporated by rotary evaporator at 40°C to a volume of 3 ml as a stock for use in bioassays and analysis by HPLC. A recirculating loop with no plants was used as a control.

### Assessment of the Root Exudates Effects on FON Conidial Germination

To determine the effect of the root exudates from the three types of plants on conidial germination, the conidial suspension was diluted to no more than 1000 conidia per mm with sterile distilled water and grown on petri dishes with 10 ml 2% water agar and 200 µl of the concentrated root exudates. Aliquots of 100 µl of the diluted suspension were spread on the plates and incubated at 28°C for 3 d. The number of colonies was counted after 3 d. A 200 µl aliquot of the condensed solution collected from the recirculating loop without plants was used in the same way and served as the control. Each treatment was replicated three times, and the value of each replication was the mean of the five plates. The effect of the root exudates on conidial germination was expressed as the relative germination impact index, which was calculated by the following formula: germination impact index = (the conidial germination number in treatment – control)/control.

### Assessment of Root Exudates Effects on FON Growth

To determine the effect of the root exudates from the three types of plants on FON growth, 5 mm-diameter plugs of fungus-containing agar excised from the margin of a 5-d-old PDA culture were inoculated on the center of petri dishes that were filled with a mixture of 10 ml 1/4 PDA and 200 µl of the concentrated root exudates and incubated at 28°C for 5 d. The colony diameters were measured in three directions on each plate after incubation for 5 d. An agar plug grown on the 1/4 PDA plate containing 200 µl of the condensed solution collected from the recirculating loop with no plants served as the control. Each treatment was replicated three times, and the value of each replication was the mean of the five plates. The effect of the root exudates on FON growth was expressed as the relative growth impact index, as calculated by the following formula: growth impact index = (the growth diameter in treatment – control)/control.

### Analysis of the Root Exudates by HPLC

Root exudates from the three types of plants were filtered through a 0.22 µm filter prior to the analysis of the root exudate profiles. The analysis was conducted by an HPLC system (Agilent 1200, USA) with an XDB-C 18 column (4.6 mm × 250 mm, Agilent, USA). The root exudate profiles were determined by the following HPLC conditions by which 11 standard phenolic compounds (gallic acid, coumaric acid, β-hydroxybenzoic acid,chlorogenic acid, vanillic acid, caffeic acid, syringic acid, ferulic acid, benzoic acid, salicylic acid and cinnamic acid) could also be completely separated. In these conditions, the mobile phase consisted of 0.1% trifluoroacetic acid (A) and acetonitrile (B) with a gradient elution of 0 min; 95% A and 5% B at a flow rate of 0.5 ml/min → 10 min; 95% A and 5% B at a rate of 1 ml/min → 20 min; 90% A and 10% B at a rate of 1 ml/min → 40 min; 65% A and 35% B at a rate of 1 ml/min → 50 min; and 65% A and 35% B at a rate of 1 ml/min. The UV detector wavelength was set at 280 nm. The column temperature was maintained at 40°C. The standard compounds were chromatographed alone and in mixtures. Retention times for the standard compounds and the major peaks in the extracts were recorded. The phenolic compounds from each fraction were identified by their retention times and the addition of standards to the samples [24].

### Assessment of Chlorogenic Acid and Caffeic Acid Effects on the Conidial Germination and Growth of FON

Because chlorogenic acid and caffeic acid were found in the root exudates of grafted-root watermelon and own-root bottle gourd but were not detected in own-root watermelon, we tested the effects of these two acids on FON conidial germination and growth. The tests were conducted as described above. The conidia were grown on 2% water agar plates with serial dilutions of either chlorogenic acid or caffeic acid for the conidial germination test, and the FON agar plugs were cultured on the 1/4 PDA plates containing serial dilutions of either chlorogenic acid or caffeic acid for the growth test. The chlorogenic acid and caffeic acid solutions were filter-sterilized (0.22 µm) before they were added to the steam-sterilized substrates. The control was the substrate without these acids. Every experimental concentration was tested with five replications per test. The germination impact index and growth impact index were also calculated according to the above formulas. The values were the means of four independent tests.

### Statistical Analysis

Analysis of variance was carried out using IMB SPSS statistics Version 20 (IBM Corporation, New York, United States). The differences in the means were determined by calculation of the least significant difference (LSD) at the 5% level.

## Results

### Effect of the Different Root Exudates on FON Conidial Germination

**Figure 2:**
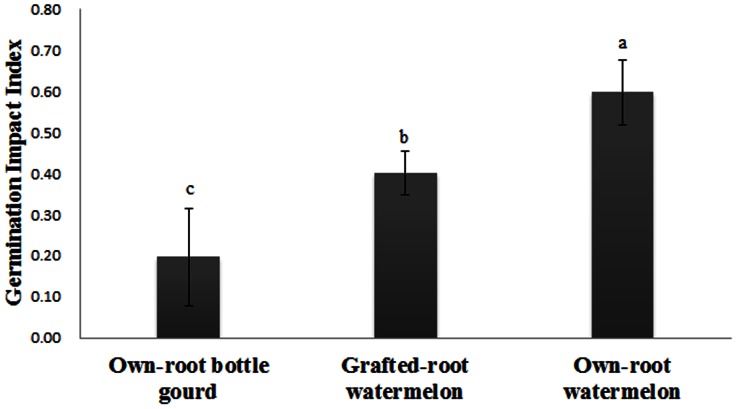
Effect of the root exudates collected from own-root bottle gourd, grafted-root watermelon and own-root watermelon on FON conidial germination. Each treatment had three replications. Bars represent standard error for three replicates. Germination impact index = (The conidial germination number in treatment – Control)/Control. The different letters represent the significance between pairs of mean values at P≤0.05 according to LSD test.

The effect of root exudates from own-root bottle gourd, grafted-root watermelon and own-root watermelon plants on FON conidial germination was tested (Fig. 2). All the germination impact indexes of the treatments were positive, which means that regardless of the type of plant, the root exudates had a stimulatory effect on FON germination to some extent. The highest germination impact index was obtained by the addition of root exudates from own-root watermelon plants into the 2% water agar, while the lowest was obtained by the addition of root exudates from own-root bottle gourd plants. These results indicate that the root exudates from own-root watermelon plants could more effectively stimulate FON conidial germination than that from the own-root bottle gourd plants. Compared with the root exudates from own-root watermelon plants, the root exudates from grafted-root watermelon plants significantly decreased the stimulatory effect on FON conidial germination. The germination impact index of the root exudates from the grafted-root watermelon plants was approximately 67% and 200% as much as that from own-root watermelon plants and own-root bottle gourd plants, respectively. These results showed that the root exudates of grafted-root watermelon could decrease FON conidial germination relative to the own-root watermelon.

### Effect of the Different Root Exudates on FON Growth

**Figure 3:**
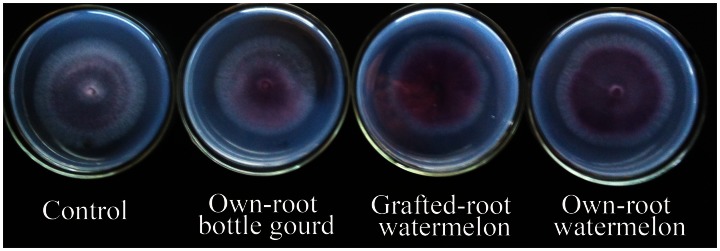
Effect of the root exudates collected from own-root bottle gourd, grafted-root watermelon and own-root watermelon on FON growth.

**Figure 4:**
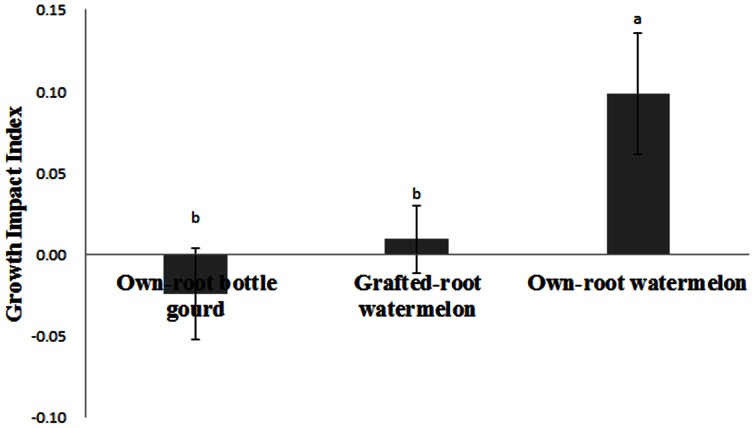
Effect of the root exudates collected from own-root bottle gourd, grafted-root watermelon and own-root watermelon on FON growth. Bars represent standard error for three replicates. Each treatment had three replications. Bars represent standard error for three replicates. Growth impact index = (The growth diameter in treatment – Control)/Control. The different letters represent the significance between pairs of mean values at P≤0.05 according to LSD test.

We also examined the effect of root exudates from own-root bottle gourd, grafted-root watermelon and own-root watermelon plants on FON growth (Fig. 3 and 4). Treatment with root exudates from own-root watermelon plants resulted in the greatest enhanced FON growth (Fig. 3), while treatment with root exudates from own-root bottle gourd had little or even negative enhancement of FON growth (Fig. 4). When the growth impact index was calculated, we found that the root exudates from grafted-root watermelon plants could significantly decrease FON growth compared with the own-root watermelon but still had a slightly greater stimulatory effect on FON growth than the control. The growth impact index after treatment with root exudates from grafted-root watermelon was less than 10 times that of the own-root watermelon.

### The Differences in the Root Exudate Profiles from these Three Types of Plants Detected by HPLC

**Figure 5:**
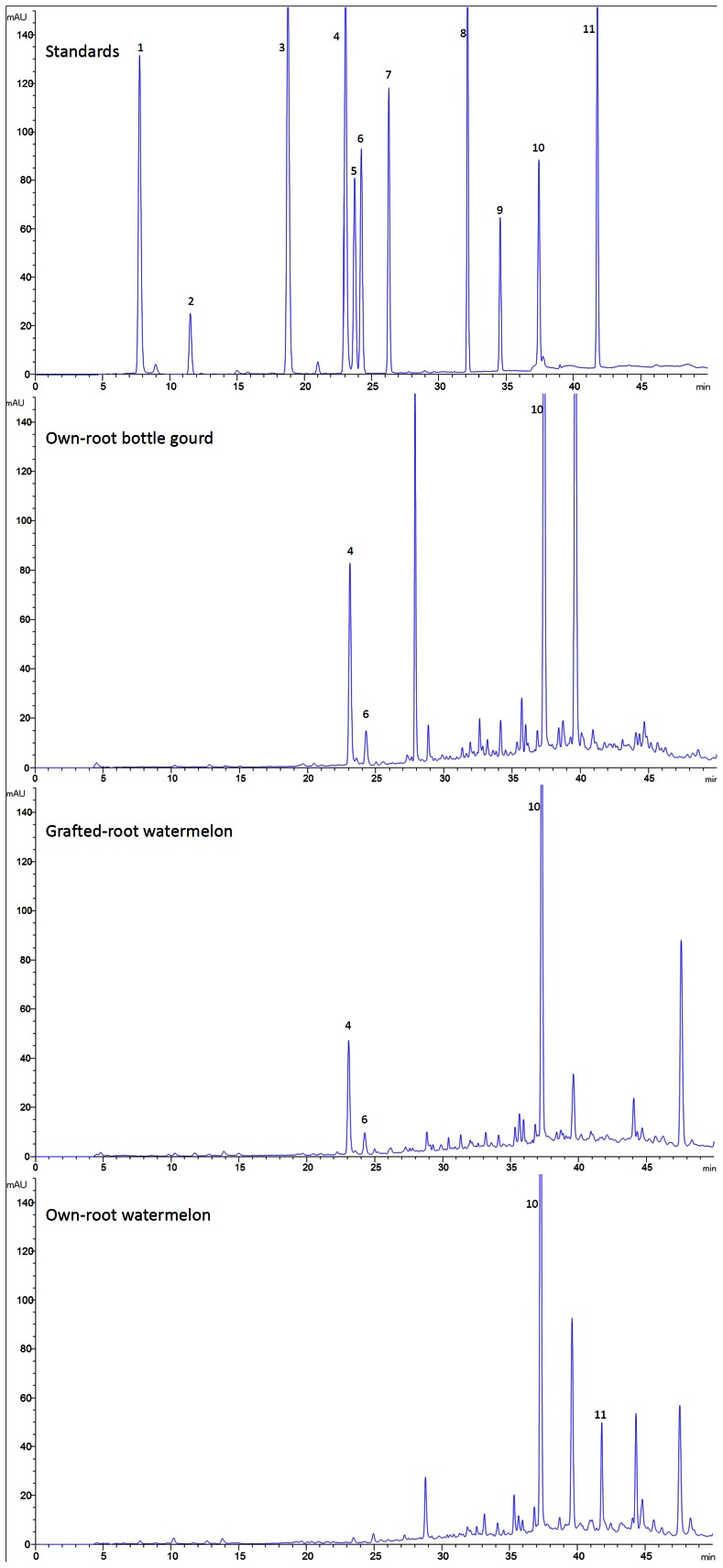
The chromatograms detected by HPLC both in standard chemicals and root exudates. The peaks from left to right in the Standards represent the following standard compounds: 1, gallic acid; 2, coumaric acid; 3, β-hydroxybenzoic acid; 4,chlorogenic acid; 5, vanillic acid; 6, caffeic acid; 7, syringic acid; 8, ferulic acid; 9, benzoic acid; 10, salicylic acid; 11, cinnamic acid. The peaks identified by HPLC in the root exudates collected from own-root bottle gourd, grafted-root watermelon and own-root watermelon were showed by the corresponding numbers.

The concentrated root exudates collected from own-root bottle gourd, grafted-root watermelon and own-root watermelon plants were analyzed by HPLC. The results showed that the chromatograms of these 3 root exudates exhibited different patterns (Fig. 5), and many peaks were detected in each root exudate. Based on comparison with the standards, we could identify some of the compounds in these 3 root exudates. As shown in Fig. 5, we found salicylic acid in all 3 root exudates, while chlorogenic acid and caffeic acid were present in the root exudates from the own-root bottle gourd and grafted-root watermelon plants but not the own-root watermelon plants, and abundant cinnamic acid was only found in own-root watermelon plant-derived root exudates.

### Effects of Chlorogenic Acid and Caffeic Acid on FON Conidial Germination

**Figure 6:**
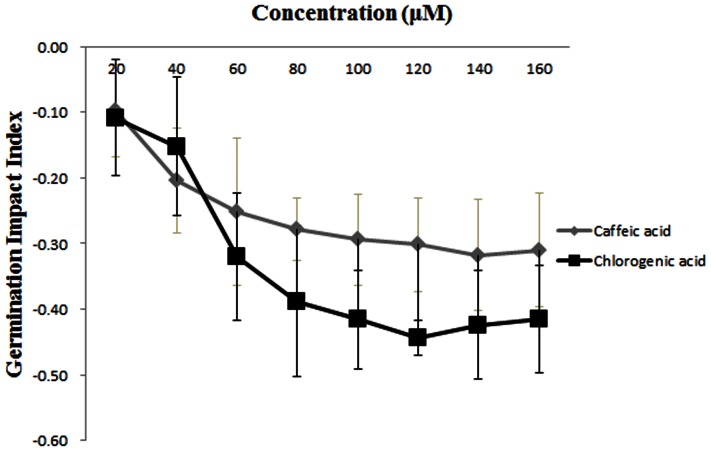
Effect of different concentrations of chlorogenic acid and caffieic acid on conidial germination of FON. Values are the means of four independent tests with standards errors shown by vertical bars. Germination impact index = (The conidial germination number in treatment – Control)/Control.

Because the root exudates from the own-root bottle gourd and grafted-root watermelon plants contained chlorogenic acid and caffeic acid, we hypothesized that they might play a role in the invasion of FON. Therefore, the effects of chlorogenic acid and caffeic acid on FON conidial germination were determined (Fig. 6). Both chlorogenic acid and caffeic acid could inhibit the germination of FON conidia, and chlorogenic acid possessed a stronger ability to inhibit FON conidial germination than caffeic acid. At concentrations between 0–80 µM, the germination impact indexes decreased rapidly with the increasing concentration of either chlorogenic acid or caffeic acid. At concentrations above 80 µM, the germination impact indexes decreased very slowly or tended toward the same level, and the germination impact index of chlorogenic acid was always lower than caffeic acid.

### Effects of Chlorogenic Acid and Caffeic Acid on FON Growth

**Figure 7:**
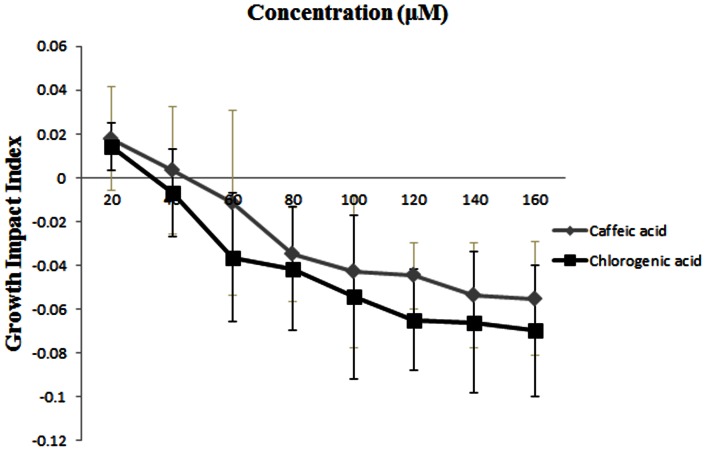
Effect of different concentrations of chlorogenic acid and caffieic acid on FON growth. Values are the means of four independent tests with standards errors shown by vertical bars. Germination impact index = (The conidial germination number in treatment – Control)/Control.

To further evaluate the effects of chlorogenic acid and caffeic acid on FON, we also examined the growth status of FON on 1/4 PDA petri dishes with different concentrations of chlorogenic acid and caffeic acid (Fig. 7). Compared with the control, chlorogenic acid and caffeic acid showed little stimulation or no effect on FON growth at concentrations between 0–40 µM. With increased concentrations of chlorogenic acid or caffeic acid, the growth impact indexes decreased (Fig. 7), which indicated that these acids could inhibit the growth of FON, and the inhibitory effect increased with increases in concentration. Similar to the effect on the germination impact index, we also found that the inhibitory effect of chlorogenic acid was always stronger than caffeic acid, as shown in Fig. 7.

## Discussion

Grafting has been proposed as a major component of an integrated management strategy for protecting plants from soil-borne diseases in areas where farm land is limited and farmers are forced to grow the same crop over successive years. Recently, own-root bottle gourds have been employed as rootstocks for commercial watermelon production in China. Many reports describing the impact of grafting on the growth and product quality of fruits and vegetables [9], [11], [17], [25] and control of soil-borne disease [10], [25], [26] have been reported. Previous research also revealed that changes in peroxidase isozymes were more pronounced in rootstocks with higher resistance [27], [28], and the isozymogram of the roots changed more significantly than that of the above-ground shoots in grafted plants [6]. We now believe that grafting not only induces metabolic alterations in scion growth but also modifies the root system directly, especially the root secretions. Here, we focused on root exudates to illustrate some aspects of the interactions between the soil-borne pathogen FOC and grafted-root or own-root plants. It has already been shown that root exudates from own-root watermelon promote FON conidial germination and FON growth [1], [2]. In the present study, the germination impact index and growth impact index were employed, which made it easy to evaluate the positive or negative effects on FON. The root exudates from own-root bottle gourds had very little stimulatory effect on FON conidial germination (Fig. 2), in fact, they inhibited FON growth (Fig. 3, 4). The root exudates of watermelon grafted onto bottle gourd rootstock could suppress FON conidial germination (Fig. 2) and growth (Fig. 3, 4) to a certain extent relative to the own-root watermelon. Consistent with previous reports, these results further confirmed that the rootstock improved the disease resistance of watermelon to *Fusarium*–mediated wilt [12], [25]. Similarly, Liu et al. [6] and Zhou et al. [16] found that mycelium growth of *Verticillium dahliae* was inhibited in the presence of root exudates from grafted eggplant and rootstock plants, and the root exudates of nongrafted eggplants enhanced mycelium growth. These data indicated that the root exudates of the watermelon plant could be changed by grafting the plant onto bottle gourd rootstock, which could result in a rhizosphere ill-suited for conidial germination and growth of the watermelon wilt disease pathogen FON.

Agronomic, physiological, and biochemical responses are altered by grafting watermelon plants onto pumpkin or other rootstocks, which is associated with higher exudation of organic acids by the roots, consequently facilitating their nutrient uptake [8], [29]. Therefore, it was reasonable to speculate that the components of root exudates were altered by grafting plants onto alternate rootstocks, preventing FON colonization of the rhizosphere. The HPLC results showed that the root exudate profiles of own-root bottle gourd, grafted-root watermelon and own-root watermelon plants were totally different (Fig. 5). The root exudates initiated and manipulated biological and physical interactions between the roots and soil microorganisms and thus played an active role in root–microbe communication [13], [14]. The resulting changes in the root exudation patterns through grafting had been believed to be at least partially involved in the altered susceptibility of plants towards soil-borne pathogens such as *F. oxysporum.* The root exudates were significantly influenced by the bottle gourd rootstock (Fig. 5), and the grafted-root watermelon was more resistant to FON attack than the own-root watermelon; the altered root exudates were able to suppress FON conidial germination (Fig. 2) and growth (Fig. 3, 4) in the rhizosphere. Our results are consistent with published reports that the root exudates released by grafted eggplants differ not only from the nongrafted eggplants but also from the tomato rootstock plants, as analyzed by gas chromatography–mass spectrometry [6], [16].

It is recognized that allelopathy plays an important role in agriculture and ecological systems. Phenolic acids are the main compounds involved in allelopathic interactions [30], and different phenolic acids in root exudates have been found to be responsible for the susceptibility or resistance of watermelon to FON [1]. In the present work, we investigated 11 phenolic acids in concentrated root exudates from own-root bottle gourd, grafted-root watermelon and own-root watermelon plants (Fig. 5). We were able to identify some phenolic acids secreted by these plants by HPLC. Salicylic acid was prevalent in all root exudates (Fig. 5). The antibiotic effect of exogenously applied salicylic acid on FON was addressed by Wu et al., who found that salicylic acid behaved as an allelochemical, greatly inhibiting FON growth and conidial formation and germination, though it stimulated mycotoxin production and the activities of hydrolytic enzymes [31]. Because salicylic acid was found in all root exudates, it is unlikely to explain the different effects of these root exudates. However, cinnamic acid was only detected in the root exudates of own-root watermelon (Fig. 5). Cinnamic acid is generally considered an allelochemical that not only significantly inhibits plant growth [32] but also significantly increases the incidence of *Fusarium*-mediated wilt in cucumber plants [33], [34]. We also deduced in a previous paper that relatively high cinnamic acid concentrations stimulate FON conidial germination at the rhizosphere level [21]. Therefore, one explanation for the decrease of FON conidial germination and FON growth by the addition of root exudates from grafted-root watermelon (Fig. 2, 3) could be that the grafting resulted in cinnamic acid secretion at such low levels that it cannot be detected by HPLC or may not secrete it at all.

In addition, chlorogenic acid and caffeic acid were found in the root exudates from own-root bottle gourd and grafted-root watermelon plants but not own-root watermelon plants. These phenolic acids might therefore have some impact on FON, so we assessed the effect of chlorogenic acid and caffeic acid on FON conidial germination (Fig. 6) and growth (Fig. 7). There is increasing evidence that chlorogenic acid is involved in host plant resistance to herbivores and insects [35], [36], as it has been observed to inhibit feeding, reduce growth and retard the developmental stages of herbivorous insects. In addition to the negative effect of chlorogenic acid on herbivores, it affects fungi, bacteria, and viruses such as *Phytophtora capsicii*, *Pseudomonas syringae* and baculovirus, as well as nuclear polyhedrosis virus [36]. In our study, FON conidial germination and growth could also be inhibited by chlorogenic acid (Fig. 6, 7). Caffeic acid exhibited the same trend as chlorogenic acid and has been reported to be fungistatic and an inhibitor of toxin production, functions that can reduce fumonisin B_1_ production by *F. verticillioides* [37] and aflatoxin production by *Aspergillus* spp. [38]. We also found that caffeic acid was able to reduce FON conidial germination and growth in vitro (Fig. 6, 7), which was consistent with the findings that caffeic acid could inhibit the growth of *Fusarium* spp. [39] and enhance the toxin (deoxynivalenol and its 15-acetylated forms) production of *F. graminearum* [40]. In this study, we also found that the inhibitory effects of chlorogenic acid and caffeic acid exhibited a dose-dependent inhibitory effect, and FON was much more susceptible to chlorogenic acid than caffeic acid (Fig. 6, 7). These data indicate that own-root bottle gourd rootstocks provide a weapon against FON in the rhizosphere by secreting chlorogenic acid and caffeic acid. This could be another reason why root exudates from grafted-root watermelon are able to decrease FON conidial germination and FON growth.

Because of the limitations of the detection system, there were many peaks in the chromatograms that were not identified. Some of them might also play a role in the grafted-root watermelon resistance to *Fusarium* (Fig. 5). A few small peaks were observed in grafted-root watermelon root exudates (Fig. 5), which could also shed light on the interactions between the watermelon scion and the bottle gourd rootstock. A more complete answer to the role of the root exudate constituents in disease-resistant rootstocks will require more advanced technology. The release of these compounds by root exudates might result in intense microbial activity (either beneficial or deleterious) and affect plant–microbe interactions in the rhizosphere and soil [13], [14]. The hypothesis that the root exudates of grafted-root watermelon might recruit more beneficial microbes than own-root watermelon will be an interesting aspect of further research into the cause of the increased resistance of grafted-root watermelon to FON.

In conclusion, this study is the first report of modification of root exudates composition in watermelon plants grafted onto bottle gourd rootstock and the effect of these changes in the relative concentrations of some phenolic acids in the root exudate on resistance to FON. The resistance of *Fusarium* wilt in grafted-root watermelon was mainly because of grafting on the root stock of a non-host of *Fusarium oxysporum* f. sp. *niveum*. However, the root exudates collected from own-root bottle gourd, grafted root watermelon and own root watermelon also gave some clue that other than the avoidance of *Fusarium oxysporum* f. sp. *niveum* by non-host root stock, the root exudates have also some contribution in the resistance to *Fusarium* wilt. The secretion of chlorogenic acid and caffeic acid and reduced secretion of cinnamic acid by grafted-root watermelon may explain the enhanced resistance to FON relative to the own-root watermelon to a certain extent. Our results are in agreement with the idea that the method of grafting onto resistant rootstocks may be used to reduce the accumulation of *F. oxysporum* in the soil in watermelon monoculture systems [12].
